# Surface display of hirame novirhabdovirus (HIRRV) G protein in *Lactococcus lactis* and its immune protection in flounder (*Paralichthys olivaceus*)

**DOI:** 10.1186/s12934-019-1195-9

**Published:** 2019-08-21

**Authors:** Lining Zhao, Xiaoqian Tang, Xiuzhen Sheng, Jing Xing, Wenbin Zhan

**Affiliations:** 1 Laboratory of Pathology and Immunology of Aquatic Animals, KLMME, Ocean University of China, 5 Yushan Road, Qingdao, 266003 China; 2 Laboratory for Marine Fisheries Science and Food Production Processes, Qingdao National Laboratory for Marine Science and Technology, Qingdao, 266071 China

**Keywords:** *Lactococcus lactis*, HIRRV glycoprotein, Surface display, Oral vaccine, Immune protection, Flounder

## Abstract

**Background:**

Hirame novirhabdovirus (HIRRV) can infect a wide range of marine and freshwater fish, causing huge economic losses to aquaculture industry. Vaccine development, especially oral vaccine, has become an effective and convenient way to control aquatic infectious diseases. HIRRV glycoprotein (G), an immunogenic viral protein is a potential vaccine candidate for prevention of the disease. Here, we aimed to construct a recombinant *Lactococcus lactis* strain expressing HIRRV-G on the cell surface as an oral vaccine to prevent HIRRV.

**Results:**

Glycoprotein gene of HIRRV was successfully cloned and expressed in *L. lactis* NZ9000 in a surface-displayed form, yielding Ll:pSLC-G. An approximately 81 kDa recombinant G protein (containing LysM anchoring motif) was confirmed by SDS-PAGE, western blotting and mass spectrometry analysis. The surface-displayed G protein was also verified by immunofluorescence and flow cytometry assays. Furthermore, to evaluate the potential of Ll:pSLC-G as oral vaccine candidate, flounders were continuously fed with commercial diet pellets coated with 1.0 × 10^9^ cfu/g of induced Ll:pSLC-G for 1 week. Four weeks later, booster vaccination was performed with the same procedure. Compared with the controls, Ll:pSLC-G elicited significantly higher levels of specific IgM against HIRRV in flounder gut mucus at the second week and in serum at the fourth week (*p* < 0.05). Meanwhile, oral immunization with Ll:pSLC-G could provide 60.7% protection against HIRRV infection and a significantly lower virus load was detected than the controls on the third day post-challenge (*p* < 0.01). Moreover, on the first day post 1-week feeding, approximately 10^4^–10^5^ recombinant *L. lactis* cells were detected in every gram of foregut, midgut and hindgut of flounder, which were mainly localized at the bottom of gut mucus layer; and on day 21, 10^2^–10^3^ *L. lactis* cells could still be recovered.

**Conclusions:**

HIRRV-G protein was successfully expressed on the surface of *L. lactis* cells, which could trigger mucosal and humoral immune response of flounder and provide considerable immune protection against HIRRV. It suggests that genetically engineered *L. lactis* expressing G protein can be employed as a promising oral vaccine against HIRRV infection.

## Background

Hirame novirhabdovirus (HIRRV), belonging to the genus *Novirhabdovirus* of *Rhabdoviridae* family, was first isolated from cultured flounder (*Paralichthys olivaceus*) and ayu (*Plecoglossus altivelis*) in Japan in 1984 [1]. Since then, it has been reported to infect several species of marine fish such as stone flounder (*Kareius bicoloratus*) [2], black seabream (*Acanthopagrus schlegeli*) [3] and spotted sea bass (*Lateolabrax maculatus*) [4], as well as two freshwater hosts, grayling (*Thymallus thymallus*) and brown trout (*Salmo trutta*) [5]. With significant morbidity and heavy economic losses, HIRRV has become a vital menace for aquaculture especially in Asia and Europe. Vaccination has become a rather important part of aquaculture, since it is considered one of the most effective disease control strategies [6]. With regard to rhabdovirus, viral glycoprotein is a spike protein responsible for virus attachment to cell receptors, and also determines the serological properties of rhabdovirus [7, 8]. So far, several vaccines against HIRRV, including subunit vaccine [9] and DNA vaccine [10, 11], were all developed based on the viral glycoprotein, which could induce a good protective immunity against HIRRV infection. Moreover, the recombinant viral hemorrhagic septicemia virus (VHSV) harboring HIRRV-G gene instead of VHSV-G gene could produce high serum neutralization titer against HIRRV but not VHSV in flounder [12]. These researches suggested that the glycoprotein of HIRRV was an attractive and promising vaccine candidate.

Oral vaccination has the advantages of being painless, less expensive and convenient to administration [13], which is particularly important and needed for aquatic animals, especially for fish fries. Oral vaccine can trigger both mucosal and systemic immune responses, which may enhance vaccine efficacy and minimize vaccine side effects by avoiding direct contact to the systemic circulation [14, 15]. Lactic acid bacteria (LAB), most of which are generally regarded as safe (GRAS), have long been used in food fermentation and preservation [16]. For nearly three decades, they have been studied as live vehicles for the delivery of antigens to mucosal sites in human, mice and chickens [17–20]. In the recent years, several LAB live vector vaccines were developed against pathogens in aquatic animals, such as infectious pancreatic necrosis virus (IPNV) in rainbow trout [21], *Streptococcus iniae* and *Edwardsiella tarda* in flounder [22, 23] and *Aeromonas veronii* in common carp [24], and showed significant immune protective effects. Therefore, it will be a promising prospect to apply lactococcal expression systems for disease prevention and control in aquaculture.

In this study, we first designed an expression cassette in *L. lactis* NZ9000 using pNZ8148 vector, and with HIRRV-G gene insertion, a recombinant *L. lactis* strain expressing G protein was constructed. After oral immunization, specific antibody responses in serum and gut mucus were analyzed in flounder model. Furthermore, the immune protective efficacies including virus load and relative percent survival (RPS) were investigated after HIRRV injection. In addition, we explored the survival and adhesion of the recombinant *L. lactis* in flounder intestine.

## Results

### Design and construction of expression cassette in *L. lactis*

**Fig. 1:**
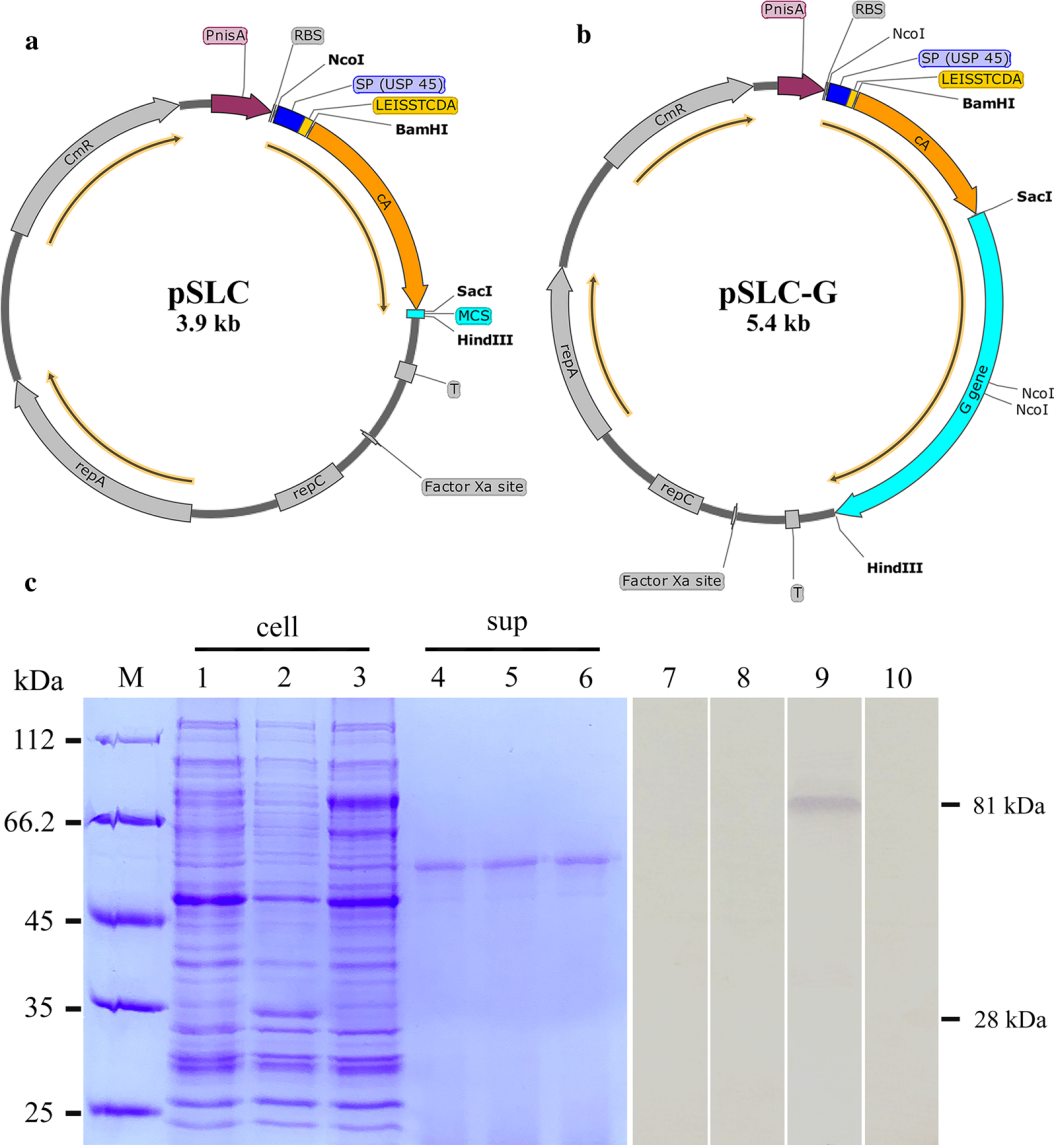
Expression of G protein on *L. lactis* NZ9000. **a**, **b** Plasmid maps of constructed expression vector (by SnapGene software), arrows indicate the length and direction of the ORFs. **a** pSLC vector, an expression cassette with MCS; **b** pSLC-G vector, expressing HIRRV-G gene. **c** SDS-PAGE and western blotting analysis of the induced recombinant *L. lactis*. M: molecular mass marker; Lane 1–3: the whole cells lysate (cell) of non-induced *L. lactis*, induced Ll:pSLC, induced Ll:pSLC-G, respectively; Lane 4–6: the corresponding culture supernatants (sup); Lane 7–9: the corresponding western blotting of non-induced *L. lactis*, induced Ll:pSLC and induced Ll:pSLC-G incubated with mouse anti-rG Pab, respectively; Line 10: western blotting of induced Ll:pSLC-G incubated with mouse negative serum

The signal peptide of Usp45 (SP_Usp45_) and the C terminus of AcmA (cA, containing three LysM motifs), as integral components for the protein secretion and surface exposure, were cloned into pNZ8148 vector, yielding pSLC. And, multiple cloning site (MCS) sequence was artificially integrated behind the cA domain, providing a pathway for insertion of the target genes. In this way, HIRRV-G gene was successfully inserted, generating pSLC-G. Under the control of P*nis*A, the open reading frame (ORF) of the constructed expression cassette was 798 bp (pSLC, Fig. 1a) or 2250 bp (pSLC-G, Fig. 1b), which was confirmed by PCR and nucleotide sequencing.

After electrotransformation, the recombinant *L. lactis* NZ9000, named Ll:pSLC and Ll:pSLC-G, were induced by nisin and then subjected to SDS-PAGE and western blotting analysis. SDS-PAGE analysis exhibited enhancement of protein bands in bacterial cell lysate samples with molecular weights of approximately 28 kDa (Ll:pSLC; Fig. 1c, lane 2) and 81 kDa (Fig. 1c, lane 3) compared to the sample before induction of Ll:pSLC-G (Fig. 1c, lane 1), which was consistent with the expectations. Nevertheless, no corresponding protein bands appeared in all supernatant samples (Fig. 1c, lanes 4–6). The 81 kDa protein band in lane 3 was submitted for mass spectrometry (MS) analysis, and the result showed that it matched 8 peptides in G protein of HIRRV with 15.4% coverage of amino acid sequences (Additional file 1: Figure S1).

### Characteristics of recombinant *L. lactis* expressing G protein

**Fig. 2:**
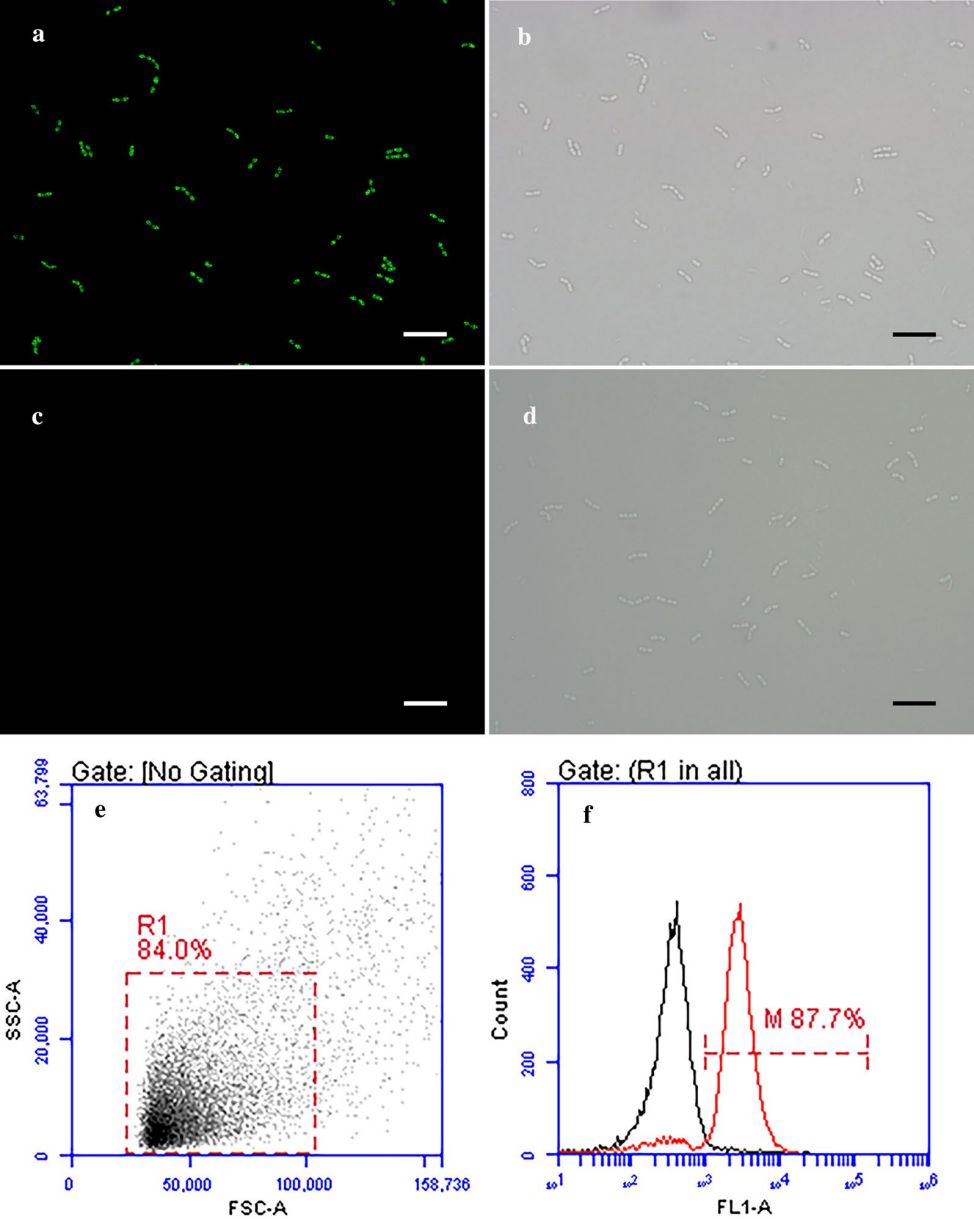
The immunofluorescence microscopy and flow cytometry analysis of recombinant Ll:pSLC-G after induction for 3 h. **a**, **c** Immunofluorescence-stained Ll:pSLC-G with mouse anti-rG Pab (**a**) and mouse negative serum (**c**); **b**, **d** bacteria were observed by interferential equipment in the same field as shown in **a** and **c**. (Bars = 10 μm). **e** *L. lactis* NZ9000 gated (R1) on a FSC/SSC dot plots; **f** fluorescence histogram of gated bacteria showing the percentages of recombinant *L. lactis* expressed G protein

HIRRV-G protein was expressed in *E. coli* Transetta with pET-28a system and mouse anti-rG polyclonal antibodies (Pab) was prepared. The results of western blotting showed that the anti-rG Pab could specifically recognize the recombinant G protein expressed by *L. lactis* (Fig. 1c, lane 9), while no stained bands appeared in the cell lysate samples of non-induced *L. lactis* and induced Ll:pSLC (Fig. 1c, lanes 7 and 8). Meanwhile, the immunofluorescence assay (IFA) was further preformed to detect whether the HIRRV-G protein could display on the bacterial cell surface, and the result showed that specific green fluorescence was observed on the surface of Ll:pSLC-G after induction (Fig. 2a). Moreover, the flow cytometry (FCM) analysis showed that the proportion of positive bacteria was more than 85% after induction for 3 h (Fig. 2f). Based on these results, we can conclude that the HIRRV-G protein was successfully expressed and displayed on the cell surface of the *L. lactis* NZ9000.

### Colonization ability of recombinant *L. lactis*

In order to detect the colonization of recombinant *L. lactis* in flounder intestine, the homogenates of foregut, midgut and hindgut were separately cultured on agar plates for 1–2 days. Typical colony characteristics, such as round, globular, white, with a smooth surface and entire edge, could be observed on the plates from the Ll:pSLC-G group, while no colonies were grown on plates from PBS (phosphate buffered saline) group. The result of colony PCR confirmed that the cultured colonies were *L. lactis* NZ9000 harboring the pSLC-G.

**Fig. 3:**
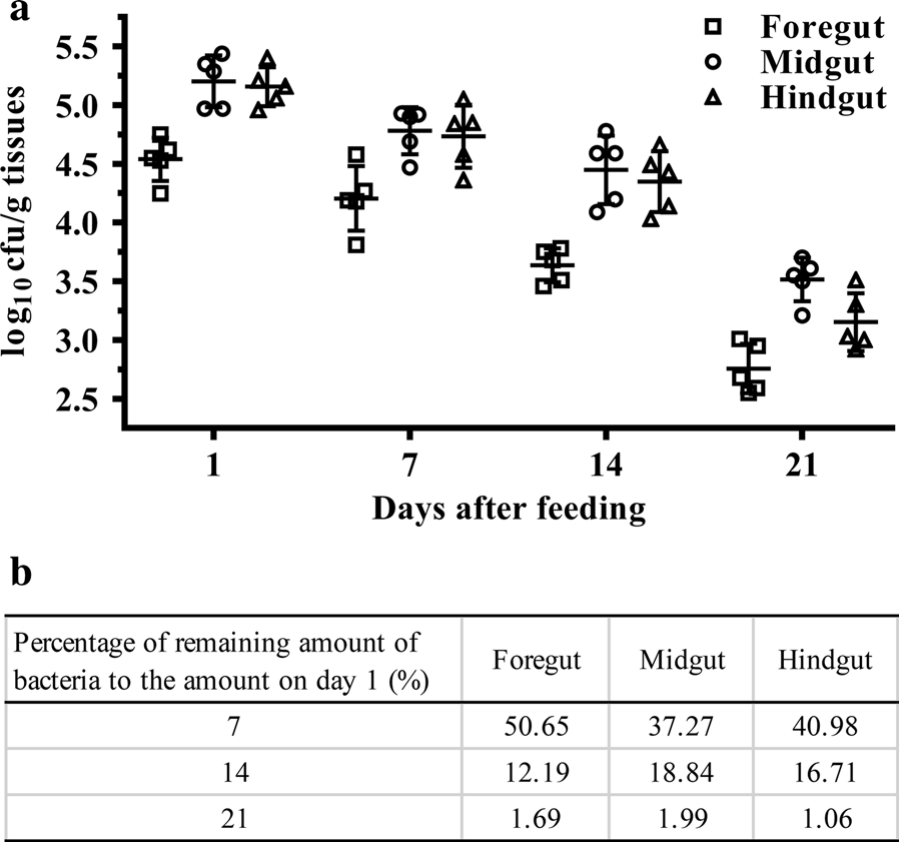
Survival and colonization of Ll:pSLC-G in the intestine of flounder. **a** Viable count of *L. lactis* isolated from different sections of flounder intestine at days 1, 7, 14 and 21 after feeding (n = 5). Mean bacterium numbers and standard deviation were represented in log_10_ scale. **b** Percentage of remaining cells on days 7, 14 and 21 compared to that on day 1

Based on plate counts, we calculated the number of recombinant *L. lactis* attached to different regions of the intestinal mucosa of flounder (Fig. 3). On the first day after oral feeding for 1 week, the calculated number of cells was approximately 10^4^–10^5^ cfu per gram homogenate of flounder intestine. Adhesion was most prominent in midgut and the number of cells was up to 1.8 × 10^5^ cfu per gram tissue. Then, the viable *L. lactis* gradually decreased with time. By the 14th day, 10^3^–10^4^ cfu per gram tissue could still be recovered from foregut, midgut and hindgut, accounting for 12.2%, 18.8% and 16.7% of the cell number on the first day, respectively. On day 21, the amount of *L. lactis* that remained adhered to the intestine tract was 10^2^–10^3^ cfu, which is less than 2% of the cell number on the first day.

**Fig. 4:**
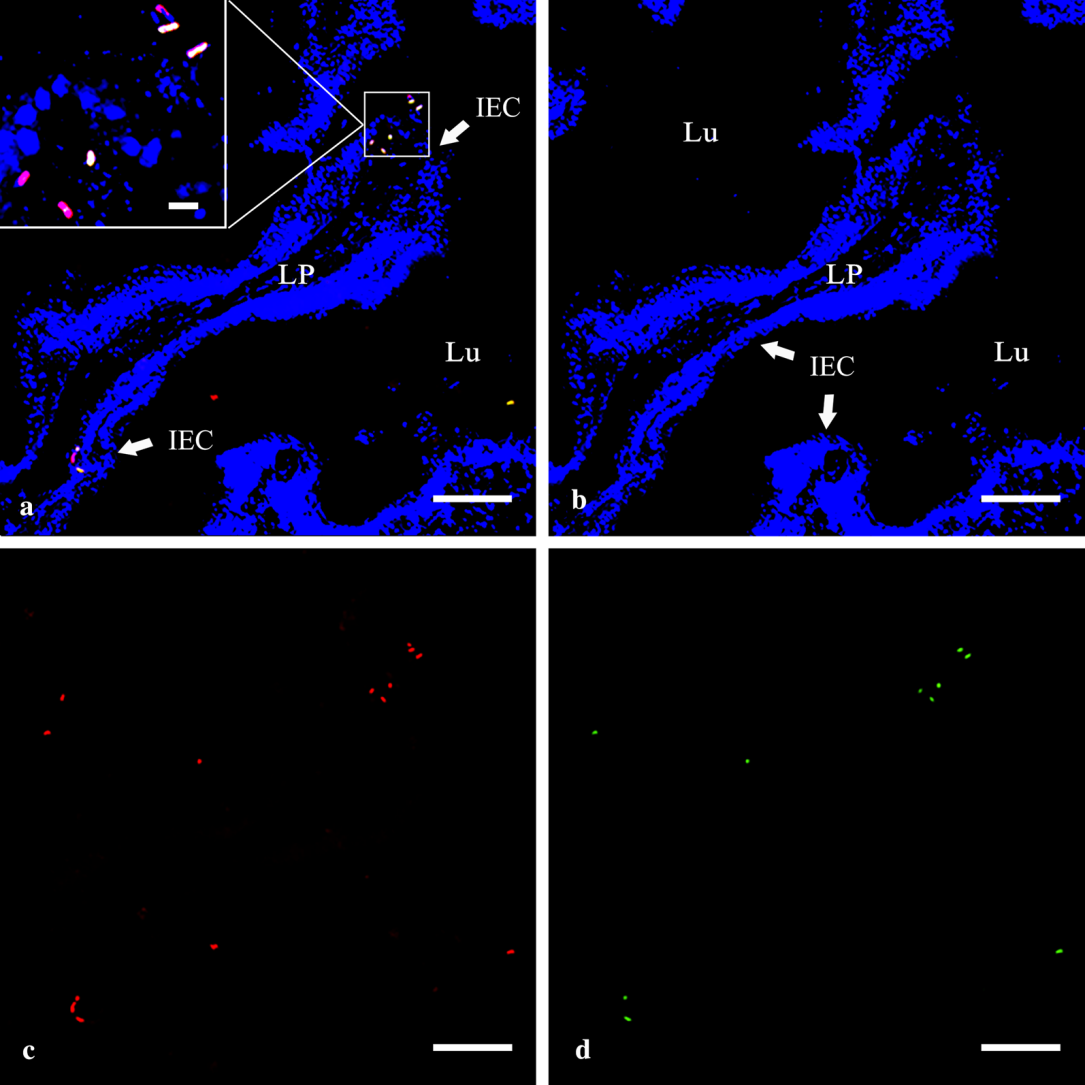
Co-staining of *L. lactis* NZ9000 and recombinant G protein in flounder intestine on 3rd day after feeding. **a** The merged picture of **b**–**d** (bar = 50 µm); the corresponding enlarged drawing was shown in the upper left of the picture, bar = 5 µm. **b** Cell nucleus were stained in blue by DAPI. **c** *L. lactis* NZ9000 was stained in red. **d** The expressed G protein was stained in green. *LP* lamina propria, *Lu* lumen; *IEC* intestinal epithelial cells

Double immunofluorescence staining was used to detect the recombinant G protein on the cell surface of *L. lactis* NZ9000 located in flounder intestine. The results showed that *L. lactis*-positive red signals were mainly localized at the bottom of gut mucus layer, near the intestinal epithelial cell layer, and some in epithelial cell layer (Fig. 4c). According to the merged picture, most of the red signals could be co-localized with G protein-positive green signals (Fig. 4a), indicating that quite a large proportion of *L. lactis* could displayed G protein. No positive signals were observed in negative control staining.

### Antibody response against HIRRV in flounder

**Fig. 5:**
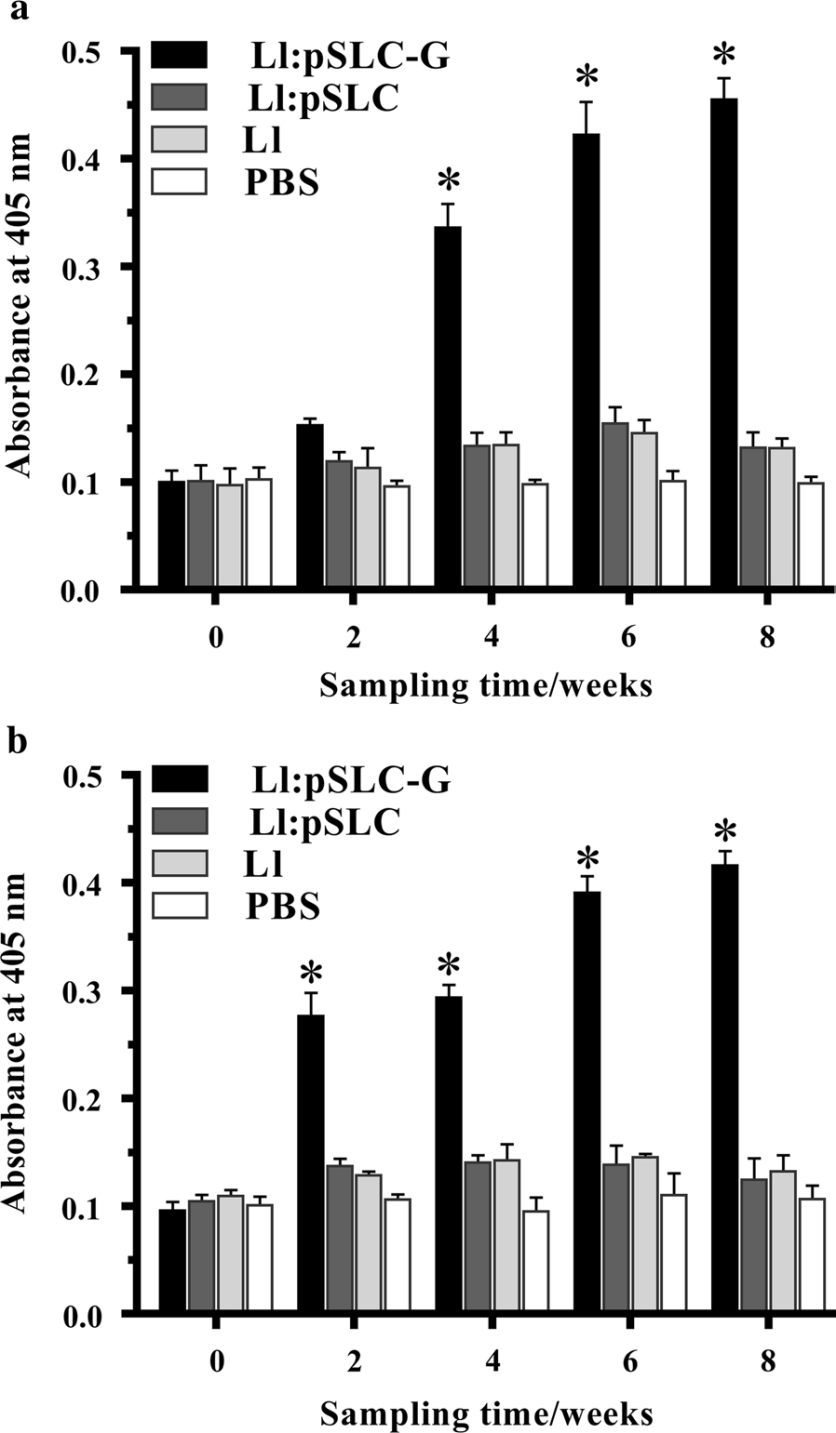
Specific IgM against HIRRV in the serum (**a**) and gut mucus (**b**) in vaccinated flounder. Results are represented as mean ± SD of five fish. Asterisks (*) on the bar represent the statistically significant difference (*p* < 0.05) as compared to PBS control

The levels of specific anti-HIRRV IgM in serum and gut mucus of the immunized flounder were determined by ELISA (Fig. 5). It showed the similar dynamic trends in serum and gut mucus samples. Compared with the controls, significantly higher level of specific IgM was elicited in Ll:pSLC-G group (*p* < 0.05). And after booster immunization, higher levels of antibodies were also observed in the flounder vaccinated with Ll:pSLC-G. By contrast, there was no significant difference among the three control groups, where flounders were fed with commercial diet pellets coated with Ll:pSLC, *L. lactis* NZ9000 (Ll) or PBS, respectively. Besides, in Ll:pSLC-G group, significant high levels of IgM were detected in gut mucus rather than serum at week 2, indicating that antibodies were elicited earlier in gut mucus.

### Immune protective efficacies against HIRRV

**Fig. 6:**
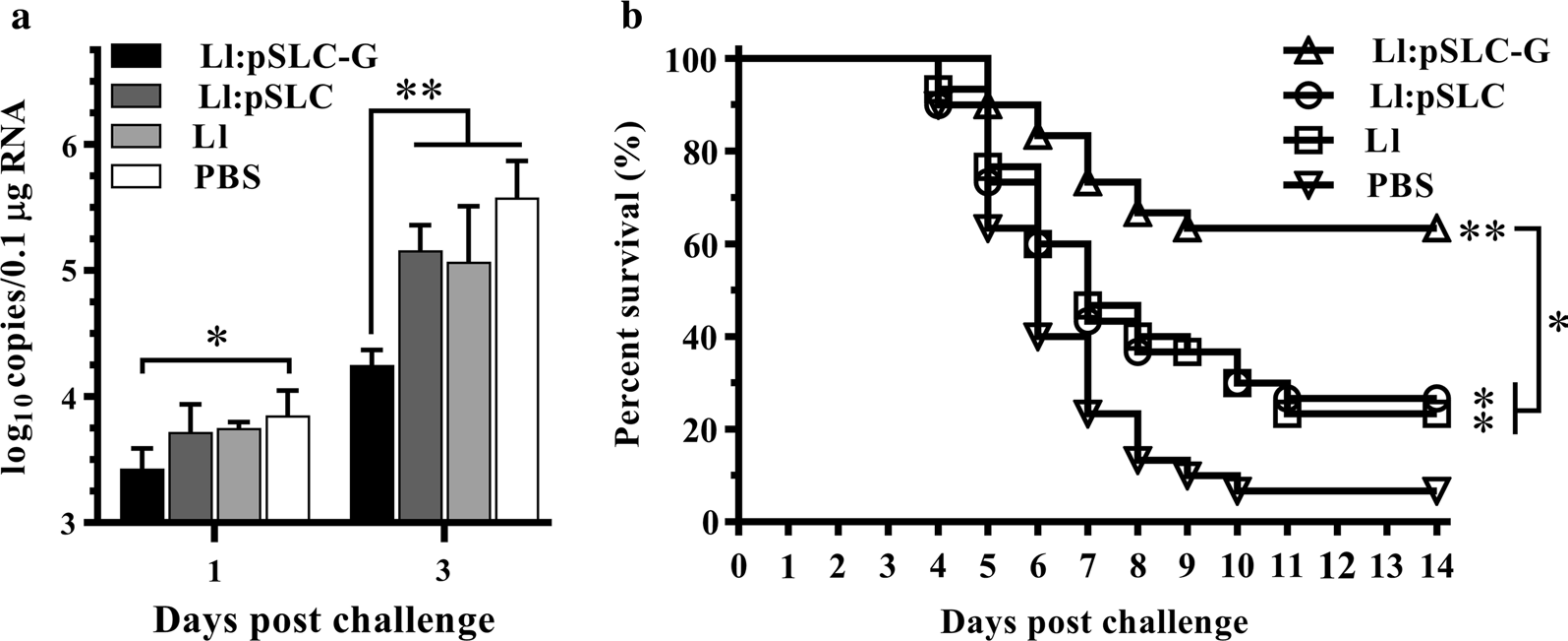
Immunoprotective effects against HIRRV elicited by oral vaccine. **a** Virus loads in spleens of vaccinated flounders post-challenge with HIRRV. Mean viral loads and standard deviation were represented in log_10_ scale. **b** The survival rates of flounder in each group, n = 30. Statistical significance was calculated after comparing PBS group by the Log-rank (Mantel-Cox) method. * indicates *p* < 0.05, ** indicates *p* < 0.01

The virus copies in spleens of challenged flounders on day 1 and 3 post-challenge were examined by qPCR, and the results were shown in Fig. 6a. In all experimental groups, the virus copies increased on day 3 compared to day 1. Compared with the controls, the fish in Ll:pSLC-G group had a significantly lower viral load on the 1st day post-challenge (*p* < 0.05), and extremely significant difference was detected on the 3rd day (*p* < 0.01). There was no statistical significant difference in virus copies among the three control groups (*p* > 0.05), but relatively low mean number of viral copies were detected in fish vaccinated with *L. lactis* NZ9000 and Ll:pSLC on the 3rd day.

The cumulative mortality of vaccinated fish after challenging with HIRRV were monitored (Fig. 6b). In PBS group, the fish began to die on 4th day post-challenge, and died quickly on 5th to 8th day; the cumulative mortality tended to be stable on day 10 at 93.3%. As for two negative groups, group Ll and Ll:pSLC, the death both occurred on day 4 to day 11, and the cumulative mortalities were 76.7% and 73.3%, respectively. In contrast, the fish vaccinated with Ll:pSLC-G showed a significantly lower mortality of 36.7% compared with the controls. The RPS of the groups Ll:pSLC-G, Ll:pSLC and Ll were 60.7%, 21.4% and 17.9%, respectively. The infected fish exhibited typical signs of HIRRV infection, such as focal hemorrhage of the skeletal muscle and fins.

## Discussion

With the extensive researches and long-term application, LAB have been proposed as mucosal delivery vehicles because of its safety and the capacity to survive in digestive tract. Due to the known complete genome, mature genetic tools and easiest genetic manipulability, *L. lactis* has been widely used for the development of candidate vaccines [25]. Till now, many different heterologous antigens have been efficiently produced in *L. lactis* [18, 26–28]. Among them, the nisin-controlled gene expression (NICE) system remains one of the most promising and widely-used inducible systems based on *nis*A promoter, whose activity depends on extracellular concentration of nisin, a small peptide produced and secreted by several strains of *L. lactis* [29]. After genetic recombination, we constructed two recombinant plasmids, pSLC and pSLC-G, which were then successfully transformed into *L. lactis* NZ9000 and induced by adding nisin. The expressed proteins could be clearly detected in SDS-PAGE and Western blotting, and the recombinant HIRRV-G protein was confirmed by MS, which indicated that the engineered G gene of HIRRV was accurately transcribed and translated into the target protein in this *L. lactis* expression system.

It is noticed that the locations of heterologous proteins produced in *L. lactis* can be intracellular, extracellular, and cell wall anchored. Displaying proteins on bacterial cell wall allows higher expression levels of protein and a better antigen exposure to the immune system [16]. Some studies demonstrated that surface-displayed strains could elicit a better immune response [30, 31]. Therefore, we chose to construct a surface display system of *L. lactis* for vaccine development. To date, the signal peptide of Usp45 (an unknown secreted protein of 45 kDa in *L. lactis*) is still the most generally used signal peptide in various expression system [16]. An interesting and valuable strategy is that synthetic LEISSTCDA peptide closely following the SP_Usp45_ may improve the secretion efficiency in *L. lactis* [32]. The cA domain of the AcmA is a very useful scaffold that can achieve cell surface display of heterologous proteins on various LABs [33]. Based on these expression elements, many proteins have been successfully expressed on the cell surface of bacteria [34–36]. In this work, according to the results of IFA and FCM analysis, the majority of cells were found to express HIRRV-G protein on the cell surface of recombinant *L. lactis* after induction. Thus, we can draw a conclusion that the HIRRV-G protein is successfully displayed on the surface of *L. lactis*, which laid the foundation for further application and effect evaluation of oral vaccine.

Rhabdoviruses consist of a helically wound ribonucleocapsid surrounded by a lipid bilayer, where glycoprotein projects outside in the form of non-covalently-bound [37]. As a spike protein, viral G protein is considered to be a crucial antigenic determinant in rhabdovirus and almost the only protein that elicits neutralization antibody, which has been sufficiently proved in infectious hematopoietic necrosis virus (IHNV) [38], another virus in the same genus. In previous studies, HIRRV-G based subunit vaccine could bring out considerable protective effects against HIRRV injection [9], and DNA vaccine constructed based on HIRRV-G gene even showed a relatively higher protection level [10]. In this work, the live vector vaccine of recombinant *L. lactis* expressing G protein exhibited a good protection of 60.7% against HIRRV infection, which is slightly lower than the effects of subunit and DNA vaccine. The small gap of the RPS value among different vaccine types might be mainly due to the difference of the amount of uptaken antigen, stimulus intensities and vaccination site. On another hand, oral vaccination offers the advantage of being easy to administer a large number of fish at one time, and it is less likely to cause immunological stress and mortality during vaccination [39]. More importantly, LAB as a functional feed additive may significantly increase weight gains and feed efficiencies [22].

Being the first barrier against the pathogens infections, mucosal immune system plays a critical role in pathogenic defense and induction of immunity. Many studies showed that oral live vector vaccine could simultaneously trigger the mucosal immune and systemic immune response of vaccinated animals [40–42]. A similar phenomenon was observed in this work: the specific IgM antibody against HIRRV were both detected in serum and gut mucus of flounders orally vaccinated with Ll:pSLC-G. Interestingly, the significant increase of antibody level was detected earlier in mucus, which indicated that mucosal immune response was quickly evoked and produced mucosal antibodies post oral vaccination. The similar result was also found in immersion vaccination in our previous work [43]. Taken together, oral vaccine can effectively trigger the mucosal and systemic immune responses, whereas mucosal immunity seems to make a quicker response.

The colonization experiment of probiotics in animals showed that colonizing bacteria can persist for different time durations in gut. In mouse model, several studies showed that LAB could exist in gut for a few days [44, 45], while some discovered that some of LAB species could not colonize the digestive tracts of man and animals, but can survive passage through the gut [28, 46]. In rainbow trout, approximately half proportions of lactobacillus were detected in the intestine at day 7 compared to the first day [40]. In the present work, we found that a portion of *L. lactis* NZ9000 has the ability to survive and adhere to the intestine in flounder and can last for at least 3 weeks. It is probably that the difference of colonizing duration may be associated with characteristics of bacteria, quantity of external supplement and the existing gastrointestinal microbiota. In addition, considerable colonization could also be achieved by continuous feeding with a high dose of probiotic bacteria for a period of time [47, 48]. Therefore, continuous or intermittent enhanced inoculation is necessary for strong and persistent immunity.

Antigen dose and vaccination scheme are crucial issues for each vaccine system [28], and have a considerable impact on immune response of vaccinated host. Our previous studies have demonstrated that the immune response is correlated to the antigen dose in injection vaccination and immersion vaccination [43, 49]. In oral vaccination, researches showed that a booster vaccination could induce a higher level of immune response in flounder and common carp [23, 41]. Therefore, a booster vaccination was conducted in this work, and the result showed that higher levels of specific IgM were produced compared to the primary immunization, indicating a prime-boost strategy is required for this oral vaccine inoculation. In order to achieve better efficacy, the immune dosage and feeding scheme need to be further optimized and explored.

## Conclusions

A surface-displayed system of *L. lactis* was constructed and the G protein of HIRRV was successfully expressed. Flounders fed with recombinant *Lactococcus* expressing G protein were triggered mucosal and systemic immune response, and exhibited a considerable protective effect against HIRRV. Hence, the genetically engineered *L. lactis* expressing G protein can be employed as a promising oral vaccine for flounder against HIRRV infection.

## Methods

### Bacterial strains, virus and fish

Bacterial strains and plasmids used in this study are listed in Additional file 1: Table S1. *E. coli* strains were grown in Luria–Bertani broth at 37 °C with vigorous shaking, while *L. lactis* strains were grown in GM17 (M17 medium supplemented with 0.5% glucose) broth at 30 °C without agitation. Agar (1.5%) was added to the medium for the plates. When necessary, ampicillin (100 μg/ml), kanamycin (50 μg/ml) and chloramphenicol (10 μg/ml) were used for *E. coli*, and chloramphenicol (10 μg/ml) and nisin (50 ng/ml) were used for *L. lactis*.

The HIRRV CNPo2015 strain, isolated from cultured flounder in 2015 [50], was used in this study. The virus was propagated in epithelioma papulosum cyprini (EPC) cell cultures at 15 °C, supplemented with 4% (v/v) fetal bovine serum (FBS), 100 IU/ml penicillin G, and 100 mg/ml streptomycin (Gibco, USA). When virus titer was around 1 × 10^6.8^ TCID_50_/100 μl, the cell cultures were harvested and centrifuged to remove cell debris. The supernatant was collected as virus supernatant for the following ELISA and challenge assay.

Specific pathogen-free flounders with body weights of 35 ± 5 g were obtained from an aquafarm in Rizhao, Shandong province of China. Before vaccination, the fish were maintained at 18–20 °C in tanks containing aerated sand-filtered seawater and fed daily with commercial diet (Great Seven, Qingdao, China) for 1 week.

### Antibodies preparation

HIRRV-G gene excluding the region of signal peptide was amplified from the virus genome with the primers G-F/G-R (Additional file 1: Table S1); the product was cloned into pET-28a vector and transformed into *E. coli* Transetta (DE3) (Transgen, Beijing, China). Following nucleotide sequencing, the confirmed clone was cultured in LB broth and induced by adding 1 mM isopropylthiogalactoside. Eight hours growth later, the cells were collected, sonicated and purified using His Trap™ HP Ni-Agarose (GE healthcare, Beijing, China) [51]. After dialysis, the protein purity was checked by SDS-PAGE and the concentration was quantified by BCA protein assay kit (Sangon Biotech, Shanghai, China). The recombinant glycoprotein was used to immunize Balb/c mice to produce the mouse anti-rG polyclonal antibodies according to the previous immunization procedure [52]. After immunization, antiserum was obtained from immunized mice, purified with protein G–agarose column (Pierce/Thermo Scientific) and characterized by western blotting.

Rabbit anti-*L. lactis* NZ9000 Pab was also prepared in this study. After cultivation, *L. lactis* cells were harvested and inactivated with PBS containing 0.5% formalin (v/v) for 72 h at 30 °C. Following reconfirmation by plate culture, inactivated *L. lactis* NZ9000 cells were washed with sterilized PBS, and the concentration was adjusted to 1.0 × 10^10^ cfu ml^−1^. New Zealand white rabbit was immunized with inactivated *L. lactis* four times, and the polyclonal antiserum was taken when the titer reached to 1:100,000–200,000. After purification, the concentration and purity of the IgG fraction were estimated using BCA protein assay kit and SDS-PAGE, respectively.

Mouse anti-flounder IgM monoclonal antibody (FIgM-Mab) was produced previously in our laboratory [52], and the dilution used in ELISA was 1: 1000.

### Construction of bacterial expression cassette

pET-32a, an expression vector for *E. coli*, was utilized for assembly of the cloning fragments. pNZ8148, an *E. coli*-*L. lactis* shuttle vector, was used as the *L. lactis* expression vector after subcloning of entire cassettes.

The signal peptide of Usp45 and the C-terminal anchor domain of AcmA gene fragments were amplified from *L. lactis* MG1363 genomic DNA using two primer pairs SP_Usp45_-F/SP_Usp45_-R and cA-F/cA-R (Additional file 1: Table S1), respectively. The purified products were digested with restriction enzymes *Nco*I/*Bam*HI (SP_Usp45_) and *Bam*HI/*Hin*dIII (cA), respectively, and ligated into the pET-32a vector one by one using T4 DNA ligase to be joined together. It is noted that the LEISSTCDA nucleotide sequence has been incorporated into the reverse primer of Usp45, which has been reported to increase the secretion of the heterologous proteins in *L. lactis* [32]. Besides, MCS sequences containing five restriction enzyme genes (*Sac*I, *Sal*I, *Eco*RI and *Hin*dIII in order of ORF) were integrated into the reverse primer of cA. The fused fragment containing SP_usp45_ and cA domain in pET-32a vector was named as SLC and is flanked by restriction enzymes *Nco*I and *Hin*dIII. Next, the connected fragment SLC was cleaved with *Nco*I/*Hin*dIII restriction enzymes and cloned into the same sites on pNZ8148 under the promoter of *nis*A, generating an expression cassette named pSLC. The recombinant vector was transformed into *E. coli* MC1061 competent cells, which were prepared by treatment with 0.1 M of cooled CaCl_2_ [53].

The amplified G gene mentioned above was inserted after cA domains in pSLC by digestion with restriction enzymes *Sac*I and *Hin*dIII, yielding pSLC-G (Fig. 1b). The DNA sequence of G gene in pSLC-G vector was confirmed via nucleotide sequencing by Tsingke Bio (Qingdao, China).

### Transformation of *L. lactis* cells and induction expression

The recombinant plasmids were transformed into *L. lactis* NZ9000 by electroporation as previously described [54] with certain modifications. For competent cell preparation, an overnight culture of *L. lactis* NZ9000 was inoculated 1:50 in fresh GM17 broth containing 1% glycine and 0.5 M sucrose and incubated at 30 °C. At early exponential phase (OD_600_ = 0.5), the culture was harvested by centrifugation (5000*g*, 4 °C and 10 min). The pellet was washed thrice with an ice-cold washing solution (5 mM KH_2_PO_4_, 2 mM MgCl_2_, 10% glycerol, 0.5 M sucrose), resuspended in 1/100 culture volume of washing solution and then electroporated immediately or stored in aliquots at − 80 °C. For electroporation, 40 μl of prepared competent cell was mixed with 1 μg of recombinant plasmid dissolved in double-distilled water, and transferred to an ice-cooled electroporation cuvette (0.2 mm). A single pulse was delivered by a Gene Pulser (Bio-Rad, USA) at 2.0 kV, 200 Ω for 4.0 ms. Immediately, the cell suspension was mixed with 900 μl of recovery medium (GM17 broth containing 0.5 M sucrose, 20 mM MgCl_2_ and 2 mM CaCl_2_) and incubated at 30 °C for 2 h. After that, 200 μl of suspension culture was spread on GM17 plate containing chloramphenicol and incubated for 2 days. Two constructed *Lactococcus lactis* were denoted Ll:pSLC and Ll:pSLC-G, respectively.

For induction, recombinant *L. lactis* cells were cultured overnight in GM17 broth with chloramphenicol and then inoculated 1:50 in fresh GM17 medium. When the OD_600_ of the culture reached 0.6, nisin was added and cultured for an additional 3 h. Then, the bacterial cells were collected by centrifugation (8000*g*, 4 °C and 5 min) for the following assays.

### Western blotting and mass spectrometry analysis

After centrifugation of the induced bacterial culture, the supernatant and cells were processed separately [55, 56]. The supernatant was filtered through 0.2-μm-size filter and proteins were precipitated by adding ice-cold 80% (w/v) TCA (16% final concentration). After washing twice with precooled acetone, the resulting pellet was dried in a vacuum centrifuge and dissolved in 50 mM NaOH. On the other hand, the cell pellets were washed twice with TES buffer (50 mM Tris–HCl, 1 mM EDTA, 25% sucrose; pH 8), resuspended in 10% volume cultures of TES buffer containing lysozyme (1 mg/ml) and subsequently incubated at 37 °C for 30 min. Then, the cell lysates were treated with ultrasound generator (Sonics VC750, USA; 750 W, 20 kHZ) on ice for 15 min (on and off cycle of 5 s at 36% amplitude). Finally, all samples were added with equal volumes of 2× loading buffer and subjected to SDS-PAGE (10% polyacrylamide). One was stained with Coomassie brilliant bule R-250, and others were transferred to polyvinyldifluoride (PVDF) membrane (Merck Millipore, USA). The PVDF membrane was blocked overnight with 4% bovine serum albumin (BSA) in PBS at 4 °C. Mouse anti-rG Pab and alkaline phosphatase (AP)-conjugated goat anti-mouse IgG (1:3000, Merck Millipore) were used as primary and secondary antibodies, respectively. Finally, the bands were stained with fresh substrate solution (100 mM NaCl, 100 mM Tris and 5 mM MgCl_2_; pH 9.5) containing nitroblue tetrazolium and 5-bromo-4-chloro-3-indolyl phosphate substrates (NBT/BCIP, Sigma), and the reaction was stopped by washing with distilled water.

According to the positive band in the PVDF membrane, the relevant band in polyacrylamide gels was excised, reduced and alkylated by 10 mM dithiothreitol and 55 mM iodoacetamide for protein identification using MS by Shanghai Applied Protein Technology (Shanghai, China).

### Immunofluorescence and FCM assays

For confirmation of the surface-displayed expression of recombinant *Lactococcus lactis*, IFA was performed as described [57]. The induced cultures of bacteria were harvested and washed twice with PBS. Cell pellets were suspended in sterile PBS containing 4% BSA and mouse anti-rG Pab, and incubated for 2 h at 37 °C. Goat anti-mouse IgG-488 conjugate (Dylight, Abbkine, China) was used as secondary antibody at a dilution of 1:500. After that, cells were washed three times with PBS. A drop of the suspension was laid onto APES coated slides and observed with fluorescence microscope. In parallel, the cell suspensions were diluted to approximately 10^6^ cells/ml and analyzed by Accuri C6 flow cytometer (BD, Accuuri™, Piscataway, NJ, USA). The primary antibody replaced with mouse negative serum was used as negative control.

### Vaccination and sampling of flounder

For oral vaccination, the induced cultures of recombinant *L. lactis* were mixed evenly with commercial diet at the final concentration of 1.0 × 10^9^ cfu/g diet. After air drying, the LAB-based feed pellets were enwrapped with sodium alginate (0.6%, *w*/*w*; food grade) and fish oil (1%, *v*/*w*) onto their surface and kept at 4 °C prior to feeding. Four groups were divided with 150 fish in each one. Experimental group of fish was fed with baits containing Ll:pSLC-G, while control group were fed with baits containing Ll:pSLC, Ll or PBS, respectively. Oral vaccination was performed at week 1 (first vaccination) and week 5 (booster vaccination) with a feeding rate of 1–2% body weight daily. Beyond those, the fish were fed regular pellet feed.

For the detection of specific antibodies, five fish from each group were randomly sacrificed to collect blood and gut mucus at weeks 0, 2, 4, 6, 8 since the beginning of feeding. Serum were separated from blood via overnight standing at 4 °C and centrifugation and then stored at − 80 °C until usage. Gut mucus was collected according to the method in previous studies [58, 59]. Briefly, gut mucus samples were collected by gently scraping the inner surface of hindgut with PBS containing 1 mM phenylmethylsulfonyl fluoride and 0.5% BSA. After vortexing and centrifugation, the supernatants were concentrated about tenfold with the same volume in all samples and stored at − 80 °C until usage.

### Adhesion study

To detect the colonization and proportion of recombinant *L. lactis* NZ9000 in fish intestine tract after one week of oral feeding, five fish in Ll:pSLC-G group and PBS group (negative control) were sacrificed on days 1, 7, 14 and 21 post feeding. Fish were starved for 24 h prior to collection and the sampling procedure was as follows. Firstly, scrub the ventral surface with 75% ethanol, extract the intestine and separate it into foregut, midgut and hindgut. Remove visible residual food particles, transfer the sections to sterile dishes and weigh. Then, wash twice with sterile PBS to rinse the passage bacteria, homogenize them and serially dilute in sterile PBS. After that, 150 µl of each sample was spread on GM17 agar plates with chloramphenicol and incubated at 30 °C for 1–2 days. When typical bacterial characteristics occurred, the bacterial colonies were counted and determined as log_10_ cfu/g tissues. Furthermore, 10 single colonies from each sample were randomly selected and subjected to colony PCR with the primers pNZ-F/pNZ-R and nisRK-F/nisRK-R (Additional file 1: Table S1), which were used to identify *L. lactis* NZ9000 and the expression cassette of recombinant plasmid, respectively. PCR products were confirmed by nucleotide sequencing.

On the other hand, the intestine samples on the third day post oral feeding were prepared for tissue cryosections according to previous description [59], and IFA was carried out. Briefly, after blocking, sections were incubated with rabbit anti-*L. lactis* NZ9000 Pab (1:2000) and mouse anti-rG Pab (1:800) for 1.5 h at 37 °C. Non-immune mouse and rabbit serum were used as negative control. After washing, goat anti-rabbit IgG-Cy3 conjugate (1:400, Merck Millipore) and goat anti-mouse IgG-488 conjugate (1:500, Dylight) were incubated for 1 h as secondary antibodies. After that, the sections were stained with DAPI for 10 min at room temperature and observed with fluorescence microscopy. All incubations were conducted in a moisture chamber in the dark, followed by three washes with PBST.

### Detection of antibodies against HIRRV by ELISA

To detect the specific IgM against HIRRV in serum and gut mucus, 100 µl of prepared virus supernatant was coated into wells of microplates (96-wells, Costar) overnight at 4 °C. After three washes with PBST (PBS containing 0.05% Tween-20), all wells were blocked with 4% BSA at 37 °C for 2 h. Thereafter, serum or mucus samples (1:20 diluted in PBS) from five flounder were incubated for 1.5 h as primary antibodies. FIgM-Mab and alkaline phosphatase-conjugated goat anti-mouse IgG were sequentially added into each well and incubated for 1 h as the secondary and third antibodies, respectively. Following three washes, 100 µl 0.1% (*w*/*v*) p-nitrophenyl phosphate (pNPP, Sigma, USA) in 50 mM carbonate-bicarbonate buffer (pH 9.8) containing 0.5 mM MgCl_2_ was added to each well and incubated at room temperature for 20 min in the dark. The reaction was stopped with 50 µl per well of 2 M NaOH and absorbance was measured with an automatic ELISA reader at 405 nm. Myeloma culture supernatant instead of FIgM-Mab was used as the negative control, and each sample was accessed in three parallels.

### Challenge and sample collection

After the last sampling at 8th week, all fish were transferred to tanks equipped with a refrigerating apparatus and adapted to 14 °C by gradual decrease of water temperature. One week later, all the fish of four experimental groups were intraperitoneally injected with 100 µl of prepared virus suspension (10^6.8^ TCID50/fish) according to the lethal dose (LD50) in flounder [50]. After challenge, thirty fish were randomly selected from each experimental group for monitoring mortality. Mortality was recorded daily for 14 d post-challenge and RPS was calculated using the following formula: (1−% mortality of vaccinated fish/% mortality of control fish) × 100. Another thirty fish were randomly selected from each experimental group for monitoring the HIRRV proliferation in the spleen of infected flounder. For sampling, three fish from each group were sacrificed on day 1 and day 3 post-injection, and the spleens were sampled and stored in Sample Protector (Takara) at − 80 °C until usage.

### Detection of HIRRV load by quantitative PCR

The absolute fluorescence quantitative PCR standard curve has been established for quantifying the G gene copies [60]. The fragment of HIRRV-G gene was cloned into the pMD19-T vector with the primers G-qF and G-qR (Additional file 1: Table S1) to construct standard plasmid. After purification and quantification, the copy number was calculated as described previously [61], and it was diluted in tenfold serial dilution series as templates for qPCR assay using Roche 480 real-time PCR system (LightCycler^®^480, USA). The 20 μl volume of the reaction mixture containing 10 μl of SYBR Green I Master, 1 μl of forward and reverse primers (10 μM), 1 μl of each diluted standard plasmid and 7 μl of RNase-free water, was subjected to the following procedure: 95 °C for 10 min, followed by 40 cycles at 95 °C for 30 s and 60 °C for 1 min. Thereafter, the threshold cycle (Ct) values were measured and standard curve equations (y = − 3.6984 x + 41.964, R^2^ = 0.9996) were produced by regression analysis of mean Ct (y) versus the log of standard plasmid copy numbers (x).

1 µg of total RNA from spleen samples was reverse transcribed into cDNA and then 2 µl of the products was used as the templates for qPCR. Following the manual above, the assay was performed in triplicate for each sample and non-infected samples were used as the negative control. After amplification, viral copies were determined by extrapolating Ct values from the standard curve and expressed as mean log_10_ copies/0.1 µg RNA.

### Statistical analysis

The comparison of antibodies production and viral copies were performed with one-way analysis of variance (ANOVA) using SPSS 20.0 software; the results were expressed as mean ± SD and the significance level was defined as *p* < 0.05. The survival rates following HIRRV challenge were compared by Log-rank (Mantel-Cox) test using GraphPad Prism 7 software.

## Supplementary information

**Additional file 1: Table S1.** Bacterial strains, plasmids and primers used in this study. **Figure S1.** Mass spectrographic analysis of the G protein expressed by *L. lactis* NZ9000. (A) The amino acid sequence of HIRRV-G protein. Eight matched peptides were underlined and two of the best matched peptides were labeled in bold. (B) Fingerprints of the two best matched peptides. (C) The matched protein information of mass spectrometric analysis.

## Data Availability

All data generated or analyzed during this study are included in this published article and its additional file.
