# Supplementary material for: Surface display of hirame novirhabdovirus (HIRRV) G protein in Lactococcus lactis and its immune protection in flounder (Paralichthys olivaceus)
Source: Microb Cell Fact. 2019 Aug 21;18:142. doi: 10.1186/s12934-019-1195-9 (PMC6704618; doi:10.1186/s12934-019-1195-9)
Supplement: Supplementary file 1 — Additional file 1: Table S1. Bacterial strains, plasmids and primers used in this study. Figure S1. Mass spectrographic analysis of the G protein expressed by L. lactis NZ9000. (A) The amino acid sequence of HIRRV-G protein. Eight matched peptides were underlined and two of the best matched peptides were labeled in bold. (B) Fingerprints of the two best matched peptides. (C) The matched protein information of mass spectrometric analysis. [file 12934_2019_1195_MOESM1_ESM.doc]

**Additional file**

**Surface display of hirame novirhabdovirus (HIRRV) G protein in** ***Lactococcus lactis* and its immune protection in flounder (*Paralichthys olivaceus*)**

Lining Zhao^1^, Xiaoqian Tang^1,2*^, Xiuzhen Sheng^1^, Jing Xing^1,2^, Wenbin Zhan^1,2^

(1) Laboratory of Pathology and Immunology of Aquatic Animals, KLMME, Ocean University of China, 5 Yushan Road, Qingdao 266003, China

(2) Laboratory for Marine Fisheries Science and Food Production Processes, Qingdao National Laboratory for Marine Science and Technology, Qingdao 266071, China

**^*^Corresponding author:** Xiaoqian Tang

**Tel:** +86-0532-82032284

**E-mails:** [tangxq@ouc.edu.cn](mailto:tangxq@ouc.edu.cn)

First author: Lining Zhao: [zln_818@163.com](mailto:zln_818@163.com)

Order of authors: Xiuzhen Sheng: [xzsheng@ouc.edu.cn](mailto:xzsheng@ouc.edu.cn)

Jing Xing: [xingjing@ouc.edu.cn](mailto:xingjing@ouc.edu.cn)

Wenbin Zhan: [wbzhan@ouc.edu.cn](mailto:wbzhan@ouc.edu.cn)

**Table S1 Bacterial strains, plasmids and primers used in this study.**

| **Strain, plasmid or primer** | **Relevant characteristics or sequence (5'-3') ^a^** | **Source or reference** |
| --- | --- | --- |
| **Strains** |  |  |
| *E. coli* DH5α | Intermediate host, plasmid free | Transgen |
| *E. coli* MC1061 | Cloning strain for the shuttle-vector pNZ8148, plasmid free | [1] |
| *E. coli* Transetta (DE3) | Expression strain for the pET-28a vector, plasmid free | Transgen |
| *L. lactis* subsp. cremoris MG1363 | Plasmid-free *Lactococcus* strain | [2] |
| *L. lactis* NZ9000 | Derivative of MG1363 carrying regulatory genes nisR and nisK on the chromosome | [3] |
| Ll:pSLC | *L. lactis* NZ9000 containing pSLC plasmid | This work |
| Ll:pSLC-G | *L. lactis* NZ9000 containing pSLC-G plasmid | This work |
| **Plasmids** |  |  |
| pET-32a | Amp^R^, commercial expression plasmid for assembly of the cloned fragments | In our lab |
| pET-28a | Kan^R^, *E. coli* expression vector with T7 promoter | In our lab |
| pNZ8148 | Cm^R^, pNZ8048 derivative; *L. lactis* expression vector with nisA promoter | Miaoling Bio |
| pSLC | pNZ8148 carrying signal peptide of Usp45 (SP_Usp45_), LEISSTCDA and anchor domain of AcmA (cA) gene with MCS sequence (SLC) fused to nisA promotor | This work |
| pSLC-G | pNZ8148 carrying SLC and G of HIRRV fused to nisA promotor | This work |
| **Primers** |  |  |
| SP_Usp45_-F | CGGCCATGGTGAAAAAAAAGATTATCTCAGCTATTTTAATGTCT (*Nco*I) | AM406671.1 |
| SP_Usp45_-R | TTCGGATCC*AGCGTCACAGGTACTGCTGATCTCTAG*AGCGTAAACACCTGACAACGGGGC (*Bam*HI) |
| cA-F | CCCGGATCCTCTTCAGCTGGAAATACTAATT (*Bam*HI) | AM406671.1 |
| cA-R | CCC*AAGCTTAGAATTCGTCGACGAGCTC*TTTTATTCGTAGATACTGACCAATT (*Hin*dIII) |
| G-F | TTCGAGCTCCAAACCATCAAGCCTGGAG (*Sac*I) | KY363350.1 |
| G-R | TTCAAGCTTACCCTCGACTGGCGAGGT (*Hin*dIII) |
| HIRRV-qF | CTTCCTGATTGTGATGTCTGCG | [4] |
| HIRRV-qR | CAACGATACTCCTGTGATTCCG |
| pNZ-F | GCATAATAAACGGCTCTGA | This work |
| pNZ-R | GCTTTATCAACTGCTGCTTT |
| nisRK-F | ACATTTCACTTCCCTTGGAT | CP002094.1 |
| nisRK-R | AAGATAGTCCAATCCCATAGTG |

^a^ Restriction enzyme sites are underlined; the nucleotide sequence of LEISSTCDA peptide in SP_Usp45_-R and the MCS sequence (containing TAA) in cA-R are italicized.

**References:**

1. Casadaban MJ, Cohen SN. Analysis of gene control signals by DNA fusion and cloning in *Escherichia coli*. J Mol Biol. 1980;138(2):179-207.

2. Gasson MJ. Plasmid complements of *Streptococcus lactis* NCDO 712 and other lactic streptococci after protoplast-induced curing. J Bacteriol. 1983;154(1):1-9.

3. Kuipers OP, de Ruyter PG, Kleerebezem M, de Vos WM. Quorum sensing-controlled gene expression in lactic acid bacteria. J Biotechnol. 1998;64(1):15-21.

4. Zhang J, Tang X, Sheng X, Xing J, Zhan W. The influence of temperature on viral replication and antiviral-related genes response in hirame rhabdovirus-infected flounder (*Paralichthys olivaceus*). Fish Shellfish Immunol. 2017;68:260-5.

**Figure S1.** Mass spectrographic analysis of the G protein expressed by *L. lactis* NZ9000. (A) The amino acid sequence of HIRRV-G protein. Eight matched peptides were underlined and two of the best matched peptides were labeled in bold. (B) Fingerprints of the two best matched peptides. (C) The matched protein information of mass spectrometric analysis.

**
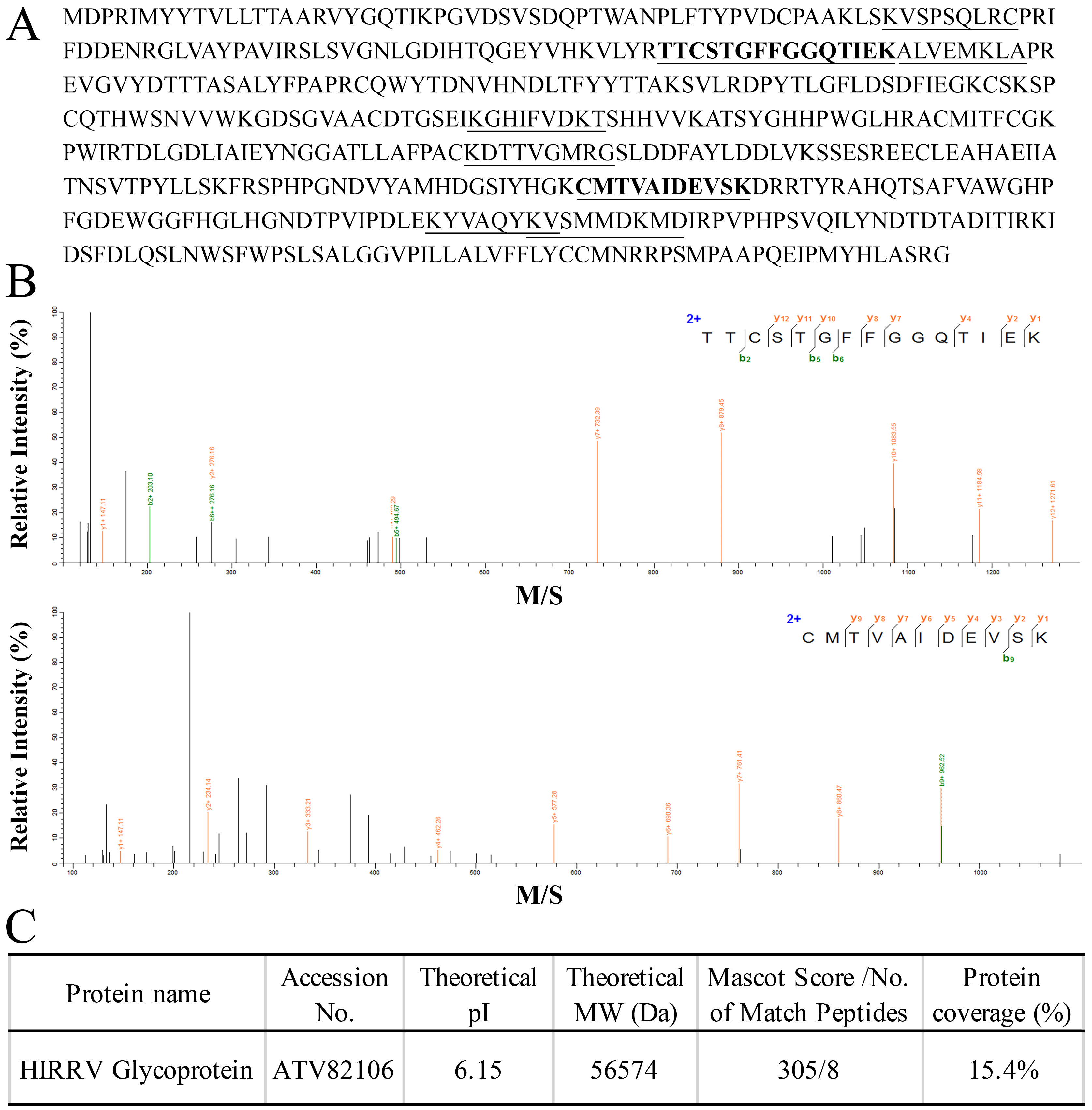
**
